# Supplementary material for: Efficient site-specific integration of large genes in mammalian cells via continuously evolved recombinases and prime editing
Source: Nat Biomed Eng. 2024 Jun 10;9(1):22–39. doi: 10.1038/s41551-024-01227-1 (PMC11754103; doi:10.1038/s41551-024-01227-1)
Supplement: Supplementary file 1 — Supplementary notes. [file 41551_2024_1227_MOESM1_ESM.pdf]

nature biomedical engineering

Supplementary information

https://doi.org/10.1038/s41551-024-01227-1

# Efficient site-specific integration of large genes in mammalian cells via continuously evolved recombinases and prime editing

In the format provided by the authors and unedited

<u>**Contents**</u>

**Supplementary Tables 1-10 |** (Provided as a separate Excel spreadsheet)

**Supplementary Note 1 |** ddPCR analysis thresholds to assess integration efficiency.

**Supplementary Note 2 |** Correlation of PASSIGE- and PASTE-mediated recombination with histone modification markers.

**Supplementary Note 3 |** Representative flow cytometry plot used to assess off-target integration in Figure 5A and Extended Data Figure 8A.

**Supplementary Note 4 |** Evaluating the validity of UDiTaS-nominated off-target candidates.

**Supplementary Note 1 | ddPCR analysis thresholds to assess integration efficiency.** The following figure shows the ddPCR plots used to assess integration efficiency at the *FANCA* locus using PASSIGE (Data shown in Figure 5A).

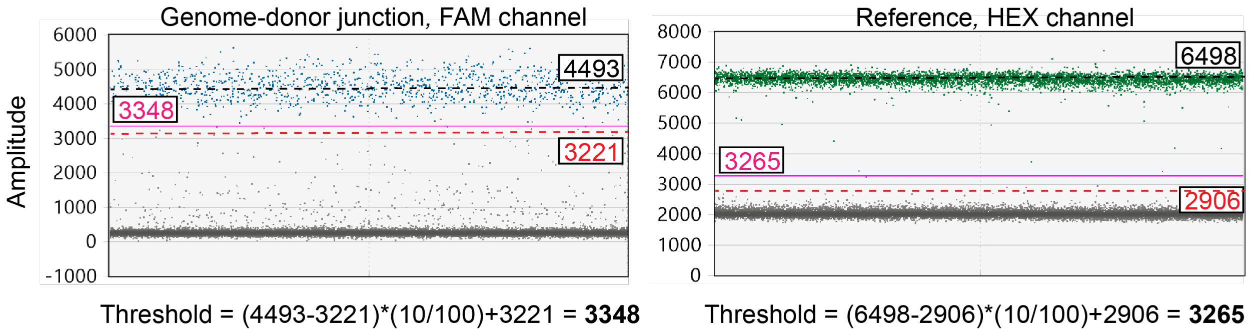


Here, the "10% rule", recommended by the ddPCR instrument manufacturer (Bio-Rad), was applied to determine a high threshold and therefore avoid false positives from plasmid-donor recombined products. In the genome-donor junction (FAM channel) a first line is drawn right above the negative cloud (3221, red dashed line). A second line is drawn around the mean of the positive cloud (4493, black dashed line). Threshold (3348, pink solid line) is then determined using the following formula:

$$\text{Threshold} = 10\% \ (\text{Amplitude of second line} - \text{Amplitude of first line}) + \text{Amplitude of first line}$$

The same process is used to determine the threshold for the reference (HEX channel). In this case, the threshold for the reference is 3265. Percent integration is determined using the following formula:

$$\% \text{ integration} = \frac{\text{Concentration of FAM channel (copies/}\mu\text{L)}}{\text{Concentration of HEX channel (copies/}\mu\text{L)}} \times 100$$

The following figure shows ddPCR plots for the FAM channel and corresponding % integration values obtained when using genome-donor junction binding probes used in this study. An *attL* or *attR* binding probe was used to assess integration efficiencies at the *GBA1* and *FANCA* loci respectively (Data shown in Figure 5a). False positives from plasmid-donor recombined products were highly reduced as the control lacking prime editor but containing eeBxb1 in both cases showed ≤0.5% integration.

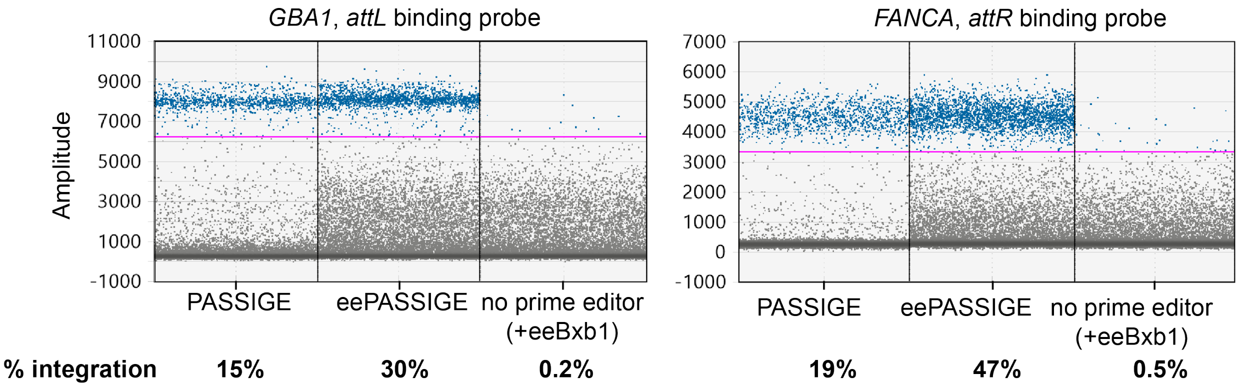

GBA1, attL binding probe
Amplitude
11000
9000
7000
5000
3000
1000
-1000
PASSIGE
eePASSIGE
no prime editor (+eeBxb1)
FANCA, attR binding probe
7000
6000
5000
4000
3000
2000
1000
0
-1000
PASSIGE
eePASSIGE
no prime editor (+eeBxb1)
% integration
15%
30%
0.2%
19%
47%
0.5%

**Supplementary Note 2 | Correlation of PASSIGE- and PASTE-mediated recombination with histone modification markers.** Recombination efficiencies of (a) eePASSIGE, (b) evoPASSIGE, (c) PASSIGE, and (d) PASTE at seven genomic loci were correlated with histone marker signals extracted and processed from ChIP-seq data deposited in ENCODE. Pearson correlation coefficient and p-value for each analysis are labeled on the scatter plot. The ChIP-seq signal of each histone modification within a 1-kb window centered around the target site was extracted and processed from HEK293T datasets. No significant correlations were observed for active chromatin markers H3K27ac and H3K4me3, $0.31 < r < 0.65$ ($p > 0.1$). For the heterochromatin-associated marker H3K9me3, no significant correlations were observed $r < -0.46$ ($p > 0.1$) except for PASTE ($p = 0.016$).

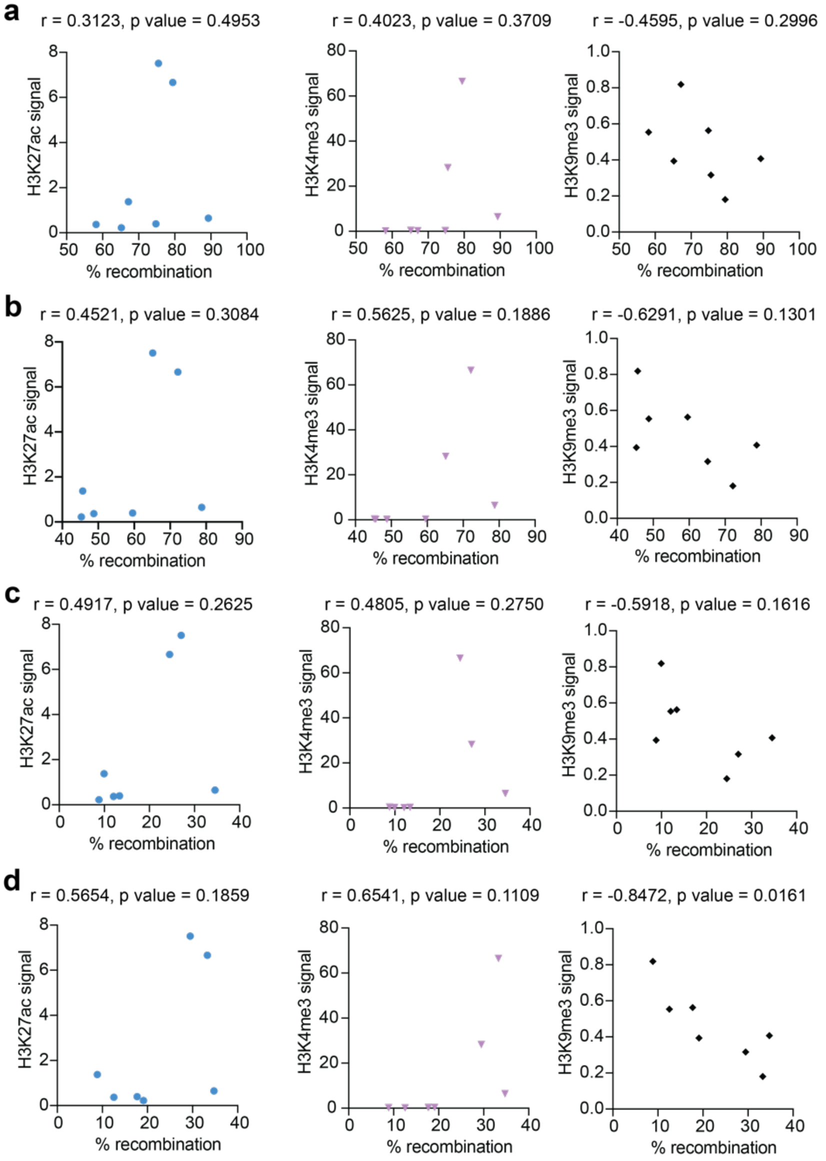

**Supplementary Note 3 | Representative flow cytometry plot used to assess off-target integration in Figure 5A and Extended Data Figure 8A.** 15,000 HEK293T cells were transfected with a recombinase variant and mCherry donor plasmid. The cells were passaged for 14 days before performing flow cytometry analysis (see more details in Methods). Cells were gated to remove dead cells (P1), then to remove doublets (P2), and finally to assess mCherry$^+$ cells from the ECD channel. Panel **a** shows an untreated sample. Panel **b** shows the histogram used to assess mCherry$^+$ cells when transfecting cells with either the dead Bxb1 negative control or eeBxb1. V1L represents mCherry$^-$ cells, and V1R represents mCherry$^+$ cells.

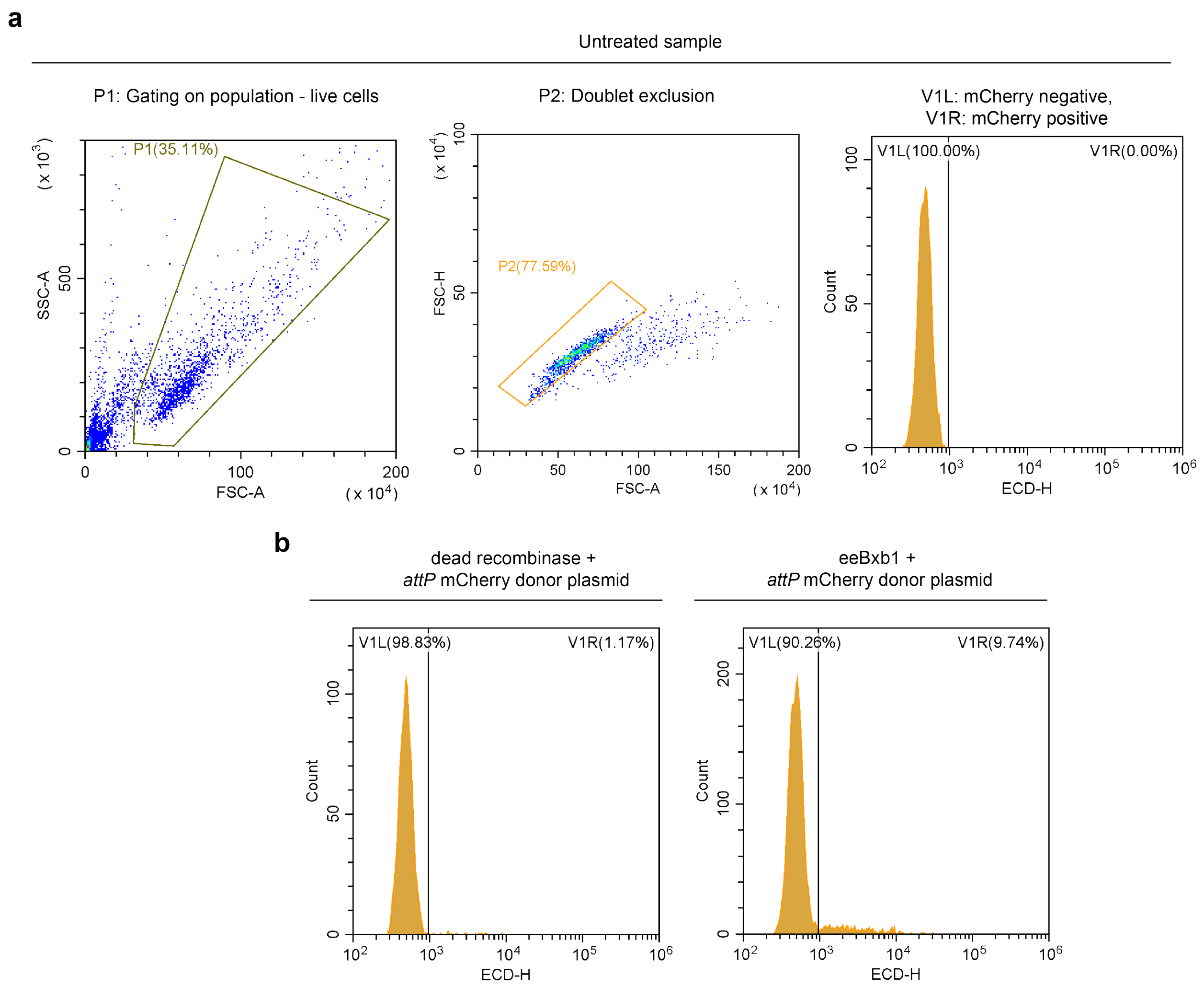

**Supplementary Note 4 | Evaluating the validity of UDiTaS-nominated off-target candidates.** A schematic of the pipeline used to prepare samples for UDiTaS is shown below in panel **a**. A detailed protocol is provided in the Methods section. In brief, Tn5 transposase was loaded with adapters to generate a transposome complex, which was subsequently used to tagment the sample DNA. Amplicons were then generated using tagmented sequence-specific and donor-specific primers and sequenced on an Illumina Miseq. Data analysis was performed as described in the Methods section.

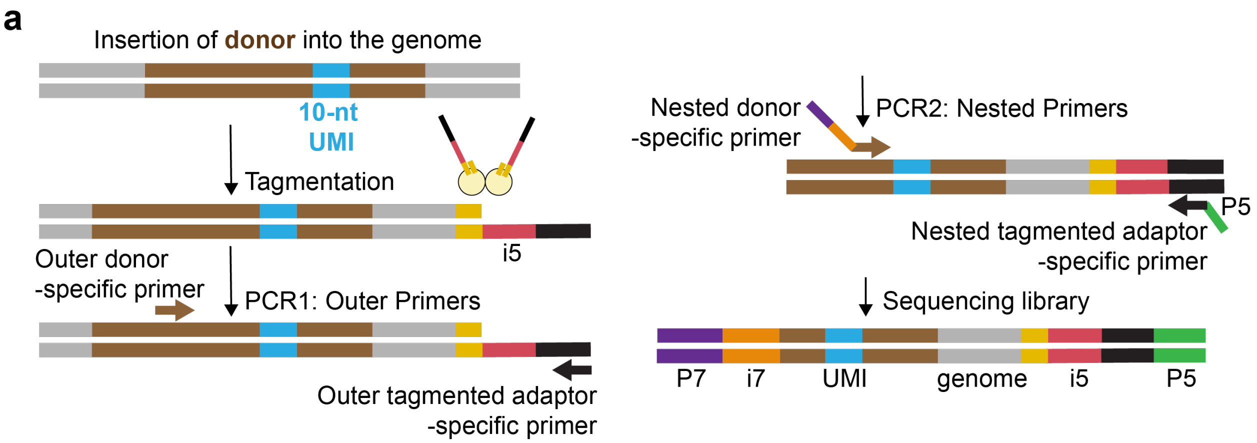


The output of the UDiTaS analysis is sequencing reads that align to both the genome and donor plasmid simultaneously. All reads from the analysis are noted in Supplementary Table S11. Based on the mechanism of recombinase-mediated integration, any real on-target or off-target event must have the genomic sequence immediately adjacent to the GT central dinucleotide, followed by the attachment (*att*) half site present in the integrated donor, as shown in panel **b**. However, in addition to these expected amplicons, UDiTaS also generated many reads in which a random sequence was present between the genome and integrated donor, as shown in panel **c** and listed in Supplementary Table S11. These sequences aligned to neither the genomic locus nor the donor plasmid.

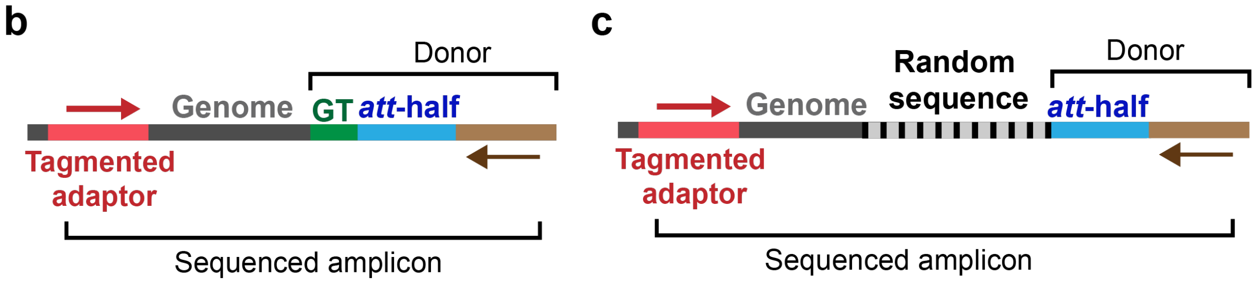

The number of amplicons containing these unexpected sequences were substantial for both PASSIGE and PASTE treated samples (Supplementary Table S11). Panel **d** below illustrates this for samples where we installed *attP* into the *AAVS1* locus. Notably, 8/10 nominated off-target sites in eePASSIGE-treated samples had an unknown sequence in between the genome and the integrated donor.

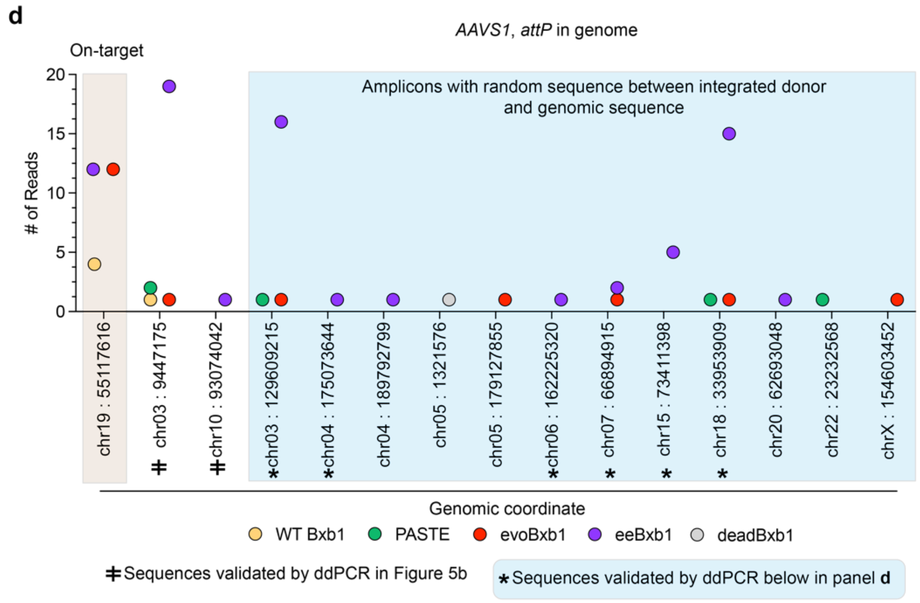


To determine the validity of these amplicons, we selected six of them for ddPCR analysis. We included all sites that appeared more than once post-deduplication and two sites that appeared only once. Additionally, for all sites, we also included a positive control in which we mixed a DNA sequence encoding the off-target sequence identified by UDiTaS with an *ACTB* reference sequence in a 1:1 ratio, so that roughly 50% of the total droplets would give a positive signal. As expected, all variants exhibited minimal integration levels (<0.08%) at the tested sites, including off-targets R_OT1 and R_OT2, which were more enriched than the on-target site in the UDiTaS analysis (panel **e**, below). These findings strongly suggest that these nominated sequences are not genuine off-target sites for integration, and further highlights the high false-positive rate associated with UDiTaS-nominated off-target candidates. For samples treated with PASSIGE variants and PASTE in which *attB* was installed into the *CCR5* locus, similar sequences emerged, albeit with lower frequencies (Supplementary Table S11).

e

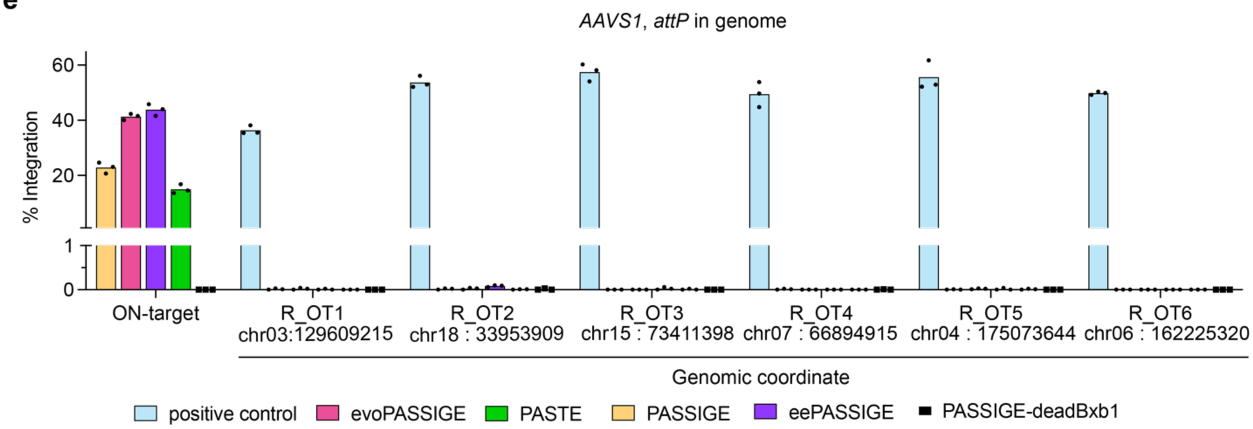


As discussed in the main text, we hypothesize that these false positives may result from template-switching during PCR, as the reverse primer used for UDiTaS sample preparation can bind to several matching DNA molecules present in cells. These molecules include the abundant donor plasmid and pegRNA-donor recombined products, as well as the on-target and any potential off-target integration sites in the genome. While it is impractical to confirm their origins for all amplicons due to the vast number of sequences present inside cells, we observed that for R_OT5, the random DNA segment between the donor and the genome, along with 5-bp of the genomic sequence fully aligned to the pegRNA-donor recombined product with a 1-bp mismatch as demonstrated in panel **f**, below. In addition to R_OT5, the random segment present in R_OT3 and R_OT6 also aligned to the Bxb1 *attR* sequence, which can only be present after recombination between DNA species containing *attP* and *attB* sequences. Since the human genome does not naturally contain an *attP* sequence, the *attR* sequence can only arise in two scenarios: at the on-target *AAVS1* locus following successful integration, and in the pegRNA-donor recombined products, suggesting that these amplicons likely originated from one of these two sources.

f

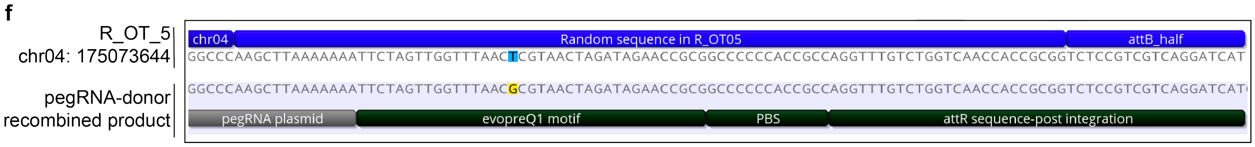


Collectively, our findings demonstrate that while UDiTaS can nominate genuine off-target integration sites (as highlighted in Extended Data Fig 8d), it also nominates many false-positive hits, which may be a consequence of template-switching during sample preparation or other mechanisms. Regardless of the cause, these findings highlight the importance of validating the authenticity of UDiTaS nominated hits using a secondary assay.
