# Supplementary material for: Efficient site-specific integration of large genes in mammalian cells via continuously evolved recombinases and prime editing
Source: Nat Biomed Eng. 2024 Jun 10;9(1):22–39. doi: 10.1038/s41551-024-01227-1 (PMC11754103; doi:10.1038/s41551-024-01227-1)
Supplement: Supplementary file 2 — Reporting Summary [file 41551_2024_1227_MOESM2_ESM.pdf]

nature portfolio

Corresponding author(s): David R. Liu

Last updated by author(s): May 7, 2024

Reporting Summary

Nature Portfolio wishes to improve the reproducibility of the work that we publish. This form provides structure for consistency and transparency in reporting. For further information on Nature Portfolio policies, see our Editorial Policies and the Editorial Policy Checklist.

Statistics

For all statistical analyses, confirm that the following items are present in the figure legend, table legend, main text, or Methods section.

n/a	Confirmed	
☐	☒	The exact sample size (*n*) for each experimental group/condition, given as a discrete number and unit of measurement
☐	☒	A statement on whether measurements were taken from distinct samples or whether the same sample was measured repeatedly
☐	☒	The statistical test(s) used AND whether they are one- or two-sided<br>*Only common tests should be described solely by name; describe more complex techniques in the Methods section.*
☐	☒	A description of all covariates tested
☐	☒	A description of any assumptions or corrections, such as tests of normality and adjustment for multiple comparisons
☐	☒	A full description of the statistical parameters including central tendency (e.g. means) or other basic estimates (e.g. regression coefficient) AND variation (e.g. standard deviation) or associated estimates of uncertainty (e.g. confidence intervals)
☐	☒	For null hypothesis testing, the test statistic (e.g. F, t, r) with confidence intervals, effect sizes, degrees of freedom and P value noted<br>*Give P values as exact values whenever suitable.*
☒	☐	For Bayesian analysis, information on the choice of priors and Markov chain Monte Carlo settings
☒	☐	For hierarchical and complex designs, identification of the appropriate level for tests and full reporting of outcomes
☐	☒	Estimates of effect sizes (e.g. Cohen's d, Pearson's r), indicating how they were calculated

Our web collection on statistics for biologists contains articles on many of the points above.

Software and code

Policy information about availability of computer code

Data collection	Illumina Miseq Control software (3.1) was used on the Illumina Miseq sequencers to collect the high-throughput sequencing data. Droplet Digital PCR data were collected using the Bio-Rad QX ONE platform.
Data analysis	CRISPResso2 was used to analyse Miseq data for quantifying %edit and %indels at genomic loci. Droplet digital PCR data were analysed using QX ONE software 1.3 Standard Edition. AmpUMI was used to deduplicate samples when noted. Code used for processing and analysing high-throughput sequencing data are available at https://github.com/pinellolab/CRISPResso2. Code used to deduplicate sequencing reads before CRISPResso2 analyis is available at http://github.com/pinellolab/AmpUMI. The Mutato code used to generate mutation tables from sequencing reads of evolved phage is available at https://hub.docker.com/r/araguram/mutato. Code used to analyse UdiTaS is available at https://github.com/Nicholas-Krasnow/integrato.

For manuscripts utilizing custom algorithms or software that are central to the research but not yet described in published literature, software must be made available to editors and reviewers. We strongly encourage code deposition in a community repository (e.g. GitHub). See the Nature Portfolio guidelines for submitting code & software for further information.

Data

Policy information about availability of data

All manuscripts must include a data availability statement. This statement should provide the following information, where applicable:

- Accession codes, unique identifiers, or web links for publicly available datasets
- A description of any restrictions on data availability
- For clinical datasets or third party data, please ensure that the statement adheres to our policy

The main data supporting the results in this study are available within the paper and its Supplementary Information. High-throughput DNA sequencing FASTQ files are available from the National Center of Biotechnology's Information Sequence Read Archive under BioProject (PRJNA1042817). Droplet digital polymerase chain reaction (ddPCR) and flow-cytometry data are available from Figshare via the identifier 10.6084/m9.figshare.24589083. Plasmids encoding evoBxb1 and eeBxb1 and a subset of pegRNA encoding plasmids are available through Addgene. Other data are available from the corresponding author on reasonable request.

Research involving human participants, their data, or biological material

Policy information about studies with human participants or human data. See also policy information about sex, gender (identity/presentation), and sexual orientation and race, ethnicity and racism.

Reporting on sex and gender	The study did not involve human research participants.
Reporting on race, ethnicity, or other socially relevant groupings	–
Population characteristics	–
Recruitment	–
Ethics oversight	–

Note that full information on the approval of the study protocol must also be provided in the manuscript.

Field-specific reporting

Please select the one below that is the best fit for your research. If you are not sure, read the appropriate sections before making your selection.

☒ Life sciences ☐ Behavioural & social sciences ☐ Ecological, evolutionary & environmental sciences

For a reference copy of the document with all sections, see nature.com/documents/nr-reporting-summary-flat.pdf

Life sciences study design

All studies must disclose on these points even when the disclosure is negative.

Sample size	Sample sizes were determined on the basis of precedents in genome-editing experiments (such as Anzalone et al., Nature 2019).
Data exclusions	No data were excluded.
Replication	The experiments described in the paper used three replicates (except for Fig. 6b, which used two replicates), and all attempts at replication were successful.
Randomization	The mammalian cells used in this study were grown under identical conditions; no randomization was used.
Blinding	The mammalian cells used in this study were grown under identical conditions; blinding was not used.

Reporting for specific materials, systems and methods

We require information from authors about some types of materials, experimental systems and methods used in many studies. Here, indicate whether each material, system or method listed is relevant to your study. If you are not sure if a list item applies to your research, read the appropriate section before selecting a response.

Materials & experimental systems

n/a	Involved in the study
☒	☐ Antibodies
☐	☒ Eukaryotic cell lines
☒	☐ Palaeontology and archaeology
☒	☐ Animals and other organisms
☒	☐ Clinical data
☒	☐ Dual use research of concern
☒	☐ Plants

Methods

n/a	Involved in the study
☒	☐ ChIP-seq
☐	☒ Flow cytometry
☒	☐ MRI-based neuroimaging

Eukaryotic cell lines

Policy information about cell lines and Sex and Gender in Research

Cell line source(s)	HEK293T (ATCC), N2A (ATCC), HuH7 (a gift from Erik Sontheimer Lab at UMMS via ATCC), Primary human fibroblasts (obtained and expanded following the declaration of Helsinki Principles at University of Minnesota Medical School), human induced pluripotent stem cells (WiCell).
Authentication	HEK293T and N2A were authenticated by the supplier using STR analysis. HuH7 cells were authenticated using ATCC's cell authentication. Primary human fibroblasts and human induced pluripotent stem cells were authenticated by the suppliers.
Mycoplasma contamination	All cell lines tested negative for mycoplasma.
Commonly misidentified lines (See ICLAC register)	No commonly misidentified cell lines were used.

Flow Cytometry

Plots

Confirm that:

☒ The axis labels state the marker and fluorochrome used (e.g. CD4-FITC).

☒ The axis scales are clearly visible. Include numbers along axes only for bottom left plot of group (a 'group' is an analysis of identical markers).

☒ All plots are contour plots with outliers or pseudocolor plots.

☒ A numerical value for number of cells or percentage (with statistics) is provided.

Methodology

Sample preparation	15,000 HEK293T cells were seeded into 96-well poly-D-lysine coated plates (Corning). 16-24 h post-seeding, cells were transfected with 100 ng of Bxb1 variant and 150 ng of an mCherry encoding donor plasmid using 0.5uL of Lipofectamine 2000 (Thermo Fisher Scientific). Cells were passaged for 2 weeks to dilute the plasmid DNA. Next, transfected cells were trypsinized, resuspended in PBS solution, and assessed for mCherry fluorescence.
Instrument	CytoFLEX S Flow Cytometer (Beckman Coulter).
Software	CytoFLEX S Flow Cytometer (Beckman Coulter) software.
Cell population abundance	The abundances of surviving single cells that were mCherry-positive were dependent on the Bxb1 variant that was transfected. These varied in the range of 0.28–12.15%. Abundances are noted in Fig. 5a and Extended data Fig. 8a.
Gating strategy	Cells were first gated (Gate A) based on forward (FSC-A) and side scattering (SSC-A) to remove dead cells and other debris. A second gate (Gate B) was used to select singlets based on FSC-H and FSC-A. Finally, mCherry-positive and mCherry-negative cells were gated (Gate C) and analysed via the ECD channel. Supplementary Note 2 highlights an example of how the cells were gated.

☒ Tick this box to confirm that a figure exemplifying the gating strategy is provided in the Supplementary Information.
